# First cases of coronavirus disease 2019 (COVID-19) in France: surveillance, investigations and control measures, January 2020

**DOI:** 10.2807/1560-7917.ES.2020.25.6.2000094

**Published:** 2020-02-13

**Authors:** Sibylle Bernard Stoecklin, Patrick Rolland, Yassoungo Silue, Alexandra Mailles, Christine Campese, Anne Simondon, Matthieu Mechain, Laure Meurice, Mathieu Nguyen, Clément Bassi, Estelle Yamani, Sylvie Behillil, Sophie Ismael, Duc Nguyen, Denis Malvy, François Xavier Lescure, Scarlett Georges, Clément Lazarus, Anouk Tabaï, Morgane Stempfelet, Vincent Enouf, Bruno Coignard, Daniel Levy-Bruhl, Investigation team

**Affiliations:** 1 Santé publique France, Direction des maladies infectieuses, Saint-Maurice, France; 2 Santé publique France, Direction des régions, Saint-Maurice, France; 3 Santé publique France, Direction des régions, Cellule Régionale Ile-de-France, Paris, France; 4 Agence Régionale de Santé Ile-de-France, Paris, France; 5 Agence Régionale de Santé Nouvelle-Aquitaine, Bordeaux, France; 6 Santé publique France, Direction des régions, Cellule Régionale Nouvelle-Aquitaine, Bordeaux, France; 7 Centre National de Référence des virus des infections respiratoires, dont la grippe, Institut Pasteur, Paris, France; 8 AP-HP, Hôpital Bichat, Service des maladies infectieuses et tropicales, Paris, France; 9 Centre Hospitalier Universitaire de Bordeaux, Service des maladies infectieuses et tropicales, Bordeaux GeoSentinel Site, Bordeaux, France; 10 UMR 1219, Université de Bordeaux, Bordeaux, France; 11 Université de Paris, IAME, INSERM, Paris, France; 12 Direction Générale de la Santé, Ministère des solidarités et de la santé, Centre opérationnel de réception et de régulation des urgences sanitaires et sociales, Paris, France; 13 Santé publique France, Direction alerte et crise, Saint-Maurice, France; 14 The members of the investigation team are listed at the end of the article

**Keywords:** coronavirus, COVID-19, SARS-CoV-2, 2019-nCov, Surveillance, contact tracing, containment, France

## Abstract

A novel coronavirus (severe acute respiratory syndrome coronavirus 2, SARS-CoV-2) causing a cluster of respiratory infections (coronavirus disease 2019, COVID-19) in Wuhan, China, was identified on 7 January 2020. The epidemic quickly disseminated from Wuhan and as at 12 February 2020, 45,179 cases have been confirmed in 25 countries, including 1,116 deaths. Strengthened surveillance was implemented in France on 10 January 2020 in order to identify imported cases early and prevent secondary transmission. Three categories of risk exposure and follow-up procedure were defined for contacts. Three cases of COVID-19 were confirmed on 24 January, the first cases in Europe. Contact tracing was immediately initiated. Five contacts were evaluated as at low risk of exposure and 18 at moderate/high risk. As at 12 February 2020, two cases have been discharged and the third one remains symptomatic with a persistent cough, and no secondary transmission has been identified. Effective collaboration between all parties involved in the surveillance and response to emerging threats is required to detect imported cases early and to implement adequate control measures.

## Background

A novel coronavirus (severe acute respiratory syndrome coronavirus 2, SARS-CoV-2) causing a cluster of respiratory infections (coronavirus disease 2019, COVID-19) in Wuhan, China, was identified on 7 January 2020 [1]. Twenty-seven patients with pneumonia had initially been reported, with an epidemiological link to a live animal market that was closed and disinfected on 1 January [1]. From 20 January, the number of notifications of cases rose dramatically, and as at 12 February 2020, 45,179 cases of SARS-CoV-2 have been confirmed, including 1,116 deaths [2]. Most of the cases (n = 44,665) were reported in 31 provinces and autonomous regions of China and 514 cases were reported in 25 other countries in Asia, Australia, Europe and North America [2]. To date, the primary source of infection remains unknown and could still be active. Human-to-human transmission was observed early after the emergence of this new virus in China and abroad, including family clusters and healthcare settings. The current outbreak dynamics strongly indicate sustained human-to-human transmission.

Strengthened surveillance of COVID-19 cases was implemented in France on 10 January 2020. The objective of the surveillance is to identify imported cases early and to prevent secondary transmission whether in the community or among healthcare workers (HCW). Investigations are carried out among contacts immediately upon illness onset and a follow-up procedure is initiated according to the evaluated level of infection risk.

Here we describe the real-time implementation of this surveillance scheme for the first three imported cases of COVID-19 identified in France, who were confirmed on 24 January 2020 in persons with a recent stay in Wuhan. Two cases were diagnosed in Paris and one in Bordeaux. We present data until 12 February on the follow-up of the cases’ contacts initiated immediately upon confirmation of infection.

## Methods

### French surveillance system

In France, according to the COVID-19 surveillance protocol, physicians suspecting a COVID-19 case have to contact immediately either the emergency hotline (SAMU-Centre 15), if the patient is seeking medical attention from a general practitioner, or a referring infectious diseases specialist at hospital level. Together, they evaluate whether the patient matches the case definition criteria for a possible case (see below). If they do, the case has to be reported immediately through a 24/7 available phone line to the Regional Health Agency (Agence régionale de santé, ARS), which informs without delay the hospital infection control teams involved in the management of the patient, the French Public Health Agency (Santé publique France, SpFrance) and the Ministry of Health.

A standardised investigation form collecting socio-demographical information, clinical details and history of exposure (history of travel to or residence in Wuhan, China or contact with a confirmed case) is completed for each possible case at regional level, in collaboration between the clinicians, the ARS and SpFrance. Data are entered into the secure web-based application Voozanoo (Epiconcept, Paris).

Possible cases have to be hospitalised, isolated and cared for in one of the 38 French referral hospitals designated by the Ministry of Health, according to the guidelines for the management of patients with Middle East respiratory syndrome (MERS) [3].

For each possible case, respiratory samples from the upper respiratory tract (nasopharyngeal swabs or aspirates) and when possible from the lower respiratory tract (bronchoalveolar lavage fluid, when indicated, or induced sputum) are collected and sent to one of the laboratories accredited to perform SARS-CoV-2-specific real-time RT-PCR. Until 27 January, only the National Reference Centre for respiratory viruses (Institut Pasteur, Paris) was able to test for the presence of the SARS-CoV-2.

### Case definition

From 17 to 29 January 2020, a possible case was defined either as a patient with a severe acute lower respiratory infection requiring admission to hospital and with a history of travel to or residence in Wuhan, China in the 14 days before symptom onset, or a patient with an acute respiratory illness whatever the severity and with a history of at-risk exposure, mainly to a confirmed case. A confirmed case was defined as a possible case with a positive SARS-CoV-2 RT-PCR on respiratory samples, performed by an accredited laboratory. Testing relied on the real-time RT-PCR procedure developed by the Charité [4] as well as on the use of real-time RT-PCR specific for the RdRp gene (four targets) designed at Institut Pasteur (RdRp-IP).

The case definition was first set up on 10 January and adapted over time. The detailed case definition used for the cases presented here as well as the most up-to-date case definition are available in the Supplement.

### Contact and co-exposure tracing

Co-exposed persons are defined as people who shared the same risks of exposure as a possible or confirmed case of COVID-19. Contact and co-exposure identification is done for all identified possible cases. Contacts are traced from the date of onset of clinical symptoms in a case. If the diagnosis of SARS-CoV-2 infection is confirmed in the index case, active surveillance of contacts/co-exposed persons is initiated immediately.

Three levels of risk of infection are defined for contacts/co-exposed persons of a possible/confirmed COVID-19 case (Table). Co-exposed persons of a confirmed case are followed-up according to the same procedure as a moderate-/high-risk contact. The follow-up procedure for the contacts/co-exposed persons differs according to the evaluation of the level of risk of infection (Table).

**Table:** Definition of a contact and follow-up procedure by level of risk of infection, COVID-19, France, January 2020

**Level of risk of infection**	**Contact definition**	**Follow-up procedure**
Negligible risk	Person who had short (< 15 min) contact with a confirmed case in public settings such as in public transportation, restaurants and shops; healthcare personnel who treated a confirmed case while wearing appropriate PPE without any breach identified.	Neither identification nor information of contacts.
Low risk	Person who had a close (within 1 m) but short (< 15 min) contact with a confirmed case, or a distant (> 1 m) but prolonged contact in public settings, or any contact in private settings that does not match with the moderate/high risk of exposure criteria.	Contacts are asked to measure their body temperature twice a day and check for clinical symptoms. In case of occurrence of symptoms like fever, cough or dyspnoea, contacts are asked to wear a surgical mask, isolate themselves and immediately contact the emergency hotline (SAMU-centre 15) indicating that they are contacts of a confirmed COVID-19 case.
Moderate/high risk	Person who had prolonged (> 15 min) direct face-to-face contact within 1 m with a confirmed case, shared the same hospital room, lived in the same household or shared any leisure or professional activity in close proximity with a confirmed case, or travelled together with a COVID-19 case in any kind of conveyance, without appropriate individual protection equipment. Healthcare personnel who treated a confirmed case without wearing appropriate PPE or with an identified breach.	In addition to the above, contacts are asked to stay at home during a 14-day period after their last contact with the confirmed case while symptomatic and to avoid contacts with the other persons living in the same household (or at least wear a surgical mask). The follow-up consists of an active follow-up through daily calls from the regional follow-up team organised by the Regional Health Agency in collaboration with Santé publique France.

COVID-19: coronavirus disease 2019; PPE: personal protective equipment.

During the initial implementation phase of the procedure, owing to the limited number of contacts involved, it was decided to also implement an active follow-up for low risk contacts.

Patients are interviewed by the clinicians, with the help of a translator if needed, who recover relevant information on their contacts since onset of clinical symptoms and the nature and intensity of exposure. The involved regional health agencies work closely with the regional entities of Santé publique France (cellules régionales) in order to implement contact tracing and follow-up. Santé publique France coordinates the surveillance at national level in liaison with the national Health Authorities.

### Ethical statement

The investigations were carried out in accordance with the General Data Protection Regulation (Regulation (EU) 2016/679 and Directive 95/46/EC) and the French data protection law (Law 78–17 on 06/01/1978 and Décret 2019–536 on 29/05/2019). Informed consent to disclosure of information relevant to this publication was obtained from the three patients confirmed with 2019-nCoV infection.

## Results

### Detected confirmed cases

Between 10 January and 24 January (period until confirmation of the first cases in France), nine possible cases were identified in France; among them, three cases were confirmed with COVID-19.

Case 1 was a 48-year-old male patient living in France. He was travelling for professional reasons in China in various cities including Wuhan when he experienced his first symptoms (fever, headaches and cough) on 16 January. He flew back to Bordeaux, France on 22 January via Shanghai, Qingdao and Paris Charles de Gaulle airports. He reported wearing a mask during the flights. He sought medical attention from a general practitioner on 23 January, where he was suspected of COVID-19, and was subsequently transferred to the regional referring hospital in Bordeaux, isolated and sampled for laboratory confirmation of SARS-CoV-2 infection. Infection was confirmed on 24 January by the National Reference Centre (Figure). Case 1 tested positive only for the E gene target when using the Charité procedure [4] and was positive for all four RdRp-IP targets with threshold cycles (Ct) in good agreement with those obtained for the E gene target.

**Figure:**
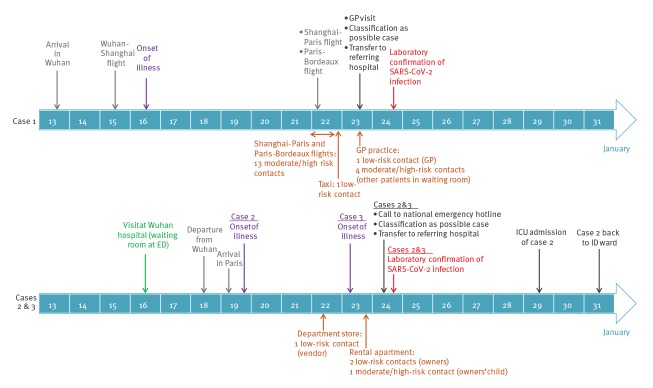
Timeline of travel, onset of illness and close contacts of confirmed cases of COVID-19, France, January 2020 (n = 3)

ED: emergency department; GP: general practitioner; ICU: intensive care unit; ID: infectious diseases.

The patient arrived in Wuhan on 13 January, did not report any visit to markets, exposure to live animals or contact with sick persons during his stay. No detailed information is available about the circumstances of exposure, apart from a visit to family members and friends on 15 January.

Case 2 was a 31-year-old Chinese male tourist who had left Wuhan on 18 January and arrived in Paris on 19 January. He developed fever, chills, fatigue, conjunctivitis and cough on 19 January. Case 3 was a 30-year-old Chinese female tourist who travelled with Case 2. She developed fever, chills, fatigue and cough on 23 January. On 24 January, they were advised by the Chinese embassy to seek medical attention at the national hotline (SAMU-centre 15) and were immediately transferred to a regional referring hospital, isolated and sampled for laboratory confirmation of COVID-19. Infection with SARS-CoV-2 was confirmed on 24 January for both of them by the National Reference Centre (Figure). Cases 2 and 3 were positive by RT-PCR for all targets of the Charité procedure [4] (RdRp Pan Sarbeco and 2019-nCov probes; E; N) as well for the four RdRp-IP targets with Ct values in good agreement with those obtained for the E gene target.

The condition of the male patient deteriorated on 29 January and he was admitted to the intensive care unit (ICU) the same day. He stayed 72 h in the ICU for non-invasive oxygen therapy and was transferred back to infectious diseases ward on 31 January.

Neither of the two cases reported any visit to markets, exposure to live animals or contact with sick persons during the 14 days before symptom onset. Both visited a hospital in Wuhan on 16 January for an unrelated medical condition in Case 3 (Figure).

As at 12 February, Case 1 was afebrile and symptomatic with a persistent cough. Cases 2 and 3 were not symptomatic any more and were discharged from hospital on 12 February.

As soon as the infection with SARS-CoV-2 was confirmed for the three cases on 24 January, this information was immediately released through a press conference held by the French Minister of Health and the Chief Medical Officer. Daily public communication on the state of the investigations around the cases was subsequently implemented by the Ministry of Health. Daily updates were also published on the SpF website.

The three cases were notified to the European Commission via the Early Warning and Response System (EWRS) on 26 January, and to the European Center for Disease Prevention and Control (ECDC) via the European Surveillance System (TESSy) on 28 January.

### Contact and co-exposition tracing

No co-exposed person was identified for Case 1. Two contacts were evaluated at low risk of infection, the taxi driver who drove the case from the airport to his home (30-min drive) and the general practitioner who took care of the patient before wearing appropriate personal protection equipment (3-min non-close contact). Seventeen contacts were evaluated at moderate/high risk of infection. Four of them shared the same waiting room in the general practitioner’s office while Case 1 was coughing, seated ca 1–1.5 m away from the case during 5–30 min. The other 13 contacts were the persons sitting in the two seats around Case 1 in the Shanghai–Paris and Paris–Bordeaux flights (Figure). They were considered at moderate risk of exposure despite the fact that Case 1 reported wearing a mask during the whole flight; this was based on the length of one of the flights (> 6 h) and the fact that it was unclear whether or not Case 1 removed his mask during short periods (e.g. meals) and kept the same mask during the whole flights. None of the contacts of the Shanghai–Paris flight were French nationals and their contact tracing was referred to their home countries’ health authorities. All other identified contacts were evaluated at negligible risk of infection because the contacts were short and/or distant in public settings and did not imply face-to-face conversations or because appropriate personal protective equipment (PPE) was worn by the healthcare personnel who took care of the patient, including those involved in the transfer from the general practitioner to the referring hospital.

Cases 2 and 3 stayed together and shared the same activities during their stay in Paris, and therefore shared the same contacts from 23 January (date of illness onset for Case 3). Three contacts were evaluated at low risk of infection: the two owners of the apartment rented by the couple and a department store employee with whom Case 2 reported a distant (> 1 m) contact during around 20 min on 22 January. The apartment owner’s child who visited Cases 2 and 3 and was hugged by them was evaluated at moderate/high risk of infection (Figure). All other identified contacts were evaluated at negligible risk of infection, as contacts were short and distant in public settings such as department stores and did not imply face-to-face conversations or because appropriate PPE was worn by the healthcare personnel who took care of the patients.

Follow-up of the identified contacts was initiated according to the COVID-19 procedure (Table). As at 2 February, two contacts have been classified as possible cases since the implementation of the follow-up: A person sitting two seats away from Case 1 during the Paris–Bordeaux flight, and therefore identified as a moderate/high risk contact, developed respiratory symptoms on 27 January and was classified as a possible case on 31 January and was subsequently excluded following negative RT-PCR results. Infection with SARS-CoV-2 was excluded on the same day. A radiology assistant who took care of both Cases 2 and 3 developed respiratory symptoms on 30 January and was classified as a possible case on 2 February. This person had been classified as at negligible risk of exposure, because she wore appropriate PPE during the whole procedure. Infection with SARS-CoV-2 was excluded on 2 February.

Follow-up of the contacts ended on 6 February. No identified contact of the three cases has been confirmed with COVID-19.

## Discussion

Specific COVID-19 surveillance has been in place in France since 10 January 2020, 3 days after the identification of the SARS-CoV-2 in China. The first three imported cases of COVID-19 in France, the first ones in Europe, were diagnosed 14 days later, on 24 January. Rapid and effective collaboration between the clinicians (general practitioners attending the cases, emergency hotline clinicians (SAMU-centre 15) and infectious diseases specialists), the National Reference Centre and the regional and national health authorities has played a crucial role in the system’s capacity to quickly detect, isolate and investigate those cases in order to implement adequate control measures. The surveillance system as well as the control measures were adapted from those implemented during past emerging infections that occurred after 2003 (severe acute respiratory syndrome (SARS), MERS, influenza A(H1N1)pdm09, Ebolavirus disease), and all involved parties were already familiar with the system, which probably favoured its responsiveness.

The case definition of a possible case in use on 24 January was slightly adapted from the one provided by the World Health Organization (WHO), based on an epidemiological link to Wuhan, China and a severe lower acute respiratory disease. It is noteworthy that the first nine possible cases identified in France, including the three confirmed cases described here, displayed mild respiratory symptoms with no sign of severity at the time of diagnosis. Increasing evidence suggests that mild clinical symptoms could be more frequent in cases of COVID-19 than with SARS-CoV and MERS-CoV [5]. Therefore, the case definition in effect on 24 January lacked sensitivity. This was counter-balanced by a tendency from the infectious diseases specialists in charge of classification of suspected cases to privilege the exposure to Wuhan over the clinical presentation in their decision. However, we cannot exclude that some COVID-19 cases remained undetected in France because of the lack of sensitivity of our case definition. The clinical criteria were expanded on 4 February to include any lower acute respiratory disease and the epidemiological criterion was extended to the whole of China. At that time, the French laboratory capacities were reinforced from one to five laboratories able to perform the diagnostics for COVID-19. Further extension to all 38 referring hospital laboratories is expected by early to mid-February 2020. Santé publique France will deploy in early February the outbreak investigation tool developed by the WHO (Go.Data [6]) in order to facilitate case data management and contact tracing at the national and local level in France.

Contact and co-exposure identification of the three confirmed cases had been initiated as soon as they were classified as possible cases, which facilitated investigations upon confirmation of COVID-19. Confirmation of the diagnosis was made in the evening of 24 January and the investigation to retrieve as exhaustively as possible contacts and co-exposed individuals and evaluate their level of risk of transmission was started immediately overnight. Complete transparency of the investigations was ensured through daily press conferences held by the French health authorities.

Although the follow-up procedure for the contacts/co-exposed persons used in France slightly differ from the ECDC and WHO guidelines [7,8], which were not available at the time of this investigation, it relies on the same general principles. Contact tracing of the passengers seated near Case 1 during the two flights Shanghai–Paris and Paris–Bordeaux was adapted from the ECDC guidelines for infectious diseases transmitted on aircraft [9]. Even though Case 1 was wearing a face mask during those flights, we could not exclude breaches and subsequent risk of transmission to the persons sitting in the two seats around him.

Because of the current uncertainties about the capacity of SARS-CoV-2 to easily spread from human to human, the decision to consider a contact as close if the case–contact distance was between 1 m and 2 m was made on a case-by-case basis, depending on the type and length of interaction. Through the extensive interviews made with the cases and their high compliance to cooperate to the investigation, we believe that the contacts most at risk have been satisfactorily identified. All of them could be rapidly contacted and informed about measures to be taken, which they all agreed to. However, some contacts were either impossible to trace back (e.g. co-travellers on public transportation) or evaluated as at negligible risk of exposure because of short and/or distant contacts (e.g. restaurant, contacts with cashiers while running errands, visiting museums), although accidental events carrying the risk of transmission on such occasions, such as an episode of cough of sneezing, cannot be ruled out.

Moreover, the contact tracing was limited to the period after onset of illness. However, should the transmission of SARS-CoV-2 occur during the asymptomatic phase, we cannot exclude that secondary transmission events initiated from the three confirmed cases remained undetected during the investigations.

Case 3 developed symptoms 4 days after her husband and 5 days after the couple had left Wuhan. The incubation period of SARS-CoV-2 is currently estimated at around 3–7 days [5,10,11]. Therefore, she may have acquired the infection from her husband, although this cannot be proved.

The active surveillance of close contacts of confirmed COVID-19 cases and the implementation of control measures, including home quarantine for those evaluated at moderate/high risk of exposure, decrease the risk of human-to-human transmission originating from imported cases and subsequently delay propagation of the virus in the general population. This allows our healthcare system to prepare for any further spread of the epidemic. Besides, the epidemiological and clinical data collected about the confirmed cases and their contacts will increase our knowledge of COVID-19.

The rapid and collaborative management of the first imported COVID-19 cases in France highlights the fact that the French healthcare system is adequately prepared to respond to such emerging diseases threats. However, this surveillance system is extremely time-consuming and requires considerable manpower. The data available on 12 February strongly suggest that human-to-human transmission of SARS-CoV-2 is frequent, with the reproduction number estimated at 2 to 3 [5,10-14]. Twenty-five countries have already reported imported cases from China, and several of them have described autochthonous transmission events [15]. In the case of further spread of SARS-CoV-2 worldwide, it would soon become impossible to detect all imported cases and trace their contacts. Especially the occurrence of large clusters in the same region would strongly impact on the local health authorities’ capacities. The surveillance objectives would then need to evolve from containing the epidemic to mitigating its medical and societal impact.

As at 12 February, the contacts of the three first confirmed cases of COVID-19 in France have been followed up for the whole 14 days follow-up time after the cases isolation. No secondary transmission event has been detected so far despite active follow-up. Given the first estimations of the SARS-CoV-2 incubation period, the probability of secondary cases originating from those three cases is negligible.

## Investigation team

Santé publique France, Direction des régions, Cellule Régionale Nouvelle Aquitaine, Bordeaux, France: Laurent Filleul, Stéphanie Vandentorren.

Santé publique France, Direction des régions, Cellule Régionale Auvergne-Rhône-Alpes, Lyon, France: Guillaume Spaccaferri.

Santé publique France, Direction des régions, Cellule Régionale Hauts-de-France, Lille, France: Hélène Prouvost.

Centre national de référence Virus des infections respiratoires, dont la grippe, Institut Pasteur, Paris, France: Mélanie Albert, Marion Barbet, Angela Brisebarre, Flora Donati, Sylvie van der Werf.

Agence Régionale de Santé Ile-de-France, Paris, France: Alexis Ardoin, Marion Dreyer, Karim Tararbit.

Agence Régionale de Santé Nouvelle-Aquitaine, Bordeaux, France: Matthieu Amodeo, Elodie Couaillier, Pascal Fabre, Daniel Habold.

Santé publique France, Direction des maladies infectieuses, Saint-Maurice, France: Didier Che.

AP-HP, Hôpital Bichat, Service des maladies infectieuses et tropicales, Paris, France: Gisèle Bendjelloul, Marine Billaudelle Lallemant, Valentine Charachon, Laurène Deconinck, Diane Descamps, Sandrine Gérard, Nadirha Houhou, Quentin Le Hingrat, Isabelle Lolom, Jean-Christophe Lucet, Annabelle Pourbaix, Simon Valayer, Yazdan Yazdanpanah.

Centre Hospitalier Universitaire de Bordeaux, Bordeaux GeoSentinel Site, Bordeaux, France: Alexandre Boyer, Benjamin Clouzeau, Xavier Combes, Arnaud Desclaux, Jean-Michel Dindart, Alexandre Duvignaud, Isabelle Garrigue, Didier Gruson, Marie-Edith Lafon, Pauline Perreau, Thierry Pistone, Maxime Poteau, Eric Tentillier.

Direction Générale de la Santé, Ministère des solidarités et de la santé, Centre opérationnel de réception et de régulation des urgences sanitaires et sociales, Paris, France: Pauline Mathieu

## Supplementary Data

123000 Supplement
