# Supplementary material for: First cases of coronavirus disease 2019 (COVID-19) in France: surveillance, investigations and control measures, January 2020
Source: Euro Surveill. 2020 Feb 13;25(6):2000094. doi: 10.2807/1560-7917.ES.2020.25.6.2000094 (PMC7029452; doi:10.2807/1560-7917.ES.2020.25.6.2000094)
Supplement: Supplement [file 20-00094_BERNARD-STOECKLIN_Supplement.pdf]

Supplementary material

This supplementary material is hosted by *Eurosurveillance* as supporting information alongside the article *"First cases of COVID-19 in France: surveillance, investigations and control measures, January 2020",* on behalf of the authors, who remain responsible for the accuracy and appropriateness of the content. The same standards for ethics, copyright, attributions and permissions as for the article apply. Supplements are not edited by *Eurosurveillance* and the journal is not responsible for the maintenance of any links or email addresses provided therein.

Case definition of COVID-19, valid from 17 to 29 January 2020

A possible case was defined as follows:

a) Any patient with clinical signs consistent with severe acute lower respiratory infection requiring admission to hospital with no other etiology that fully explains the clinical presentation AND with a history of travel to or residence in the city of Wuhan, Hubei Province, China, in the 14 days prior to symptom onset.

b) Any patient with any acute respiratory illness, whatever the severity, AND with history of at least one of the following exposures in the 14 days prior to illness onset:
   - close contact with a confirmed case of COVID-2019, while symptomatic;
   - having shared the same risks of exposure as a confirmed case of COVID-2019 (i.e. same history of travel to or residence in the city of Wuhan, Hubei Province, China);
   - having worked or attended a health care facility where patients with COVID-2019 have been reported;
   - having visited or worked in a live animal market in Wuhan, Hubei Province, China.

c) Any patient with severe acute respiratory infection for whom an etiology that fully explains the clinical presentation has been initially identified, who develops an unexpected clinical course deterioration AND with a history of travel to or residence in the city of Wuhan, Hubei Province, China, in the 14 days prior to symptom onset.

A **confirmed case** was defined as a possible case with a positive SARS-CoV-2 RT-PCR on respiratory samples, performed by an accredited laboratory.

Case definition of COVID-19, valid from 4 February 2020

A possible case was defined as follows:

a) Any patient with clinical signs consistent with a lower acute respiratory infection with fever or feeling of fever, whatever the severity, AND with a history of travel to or residence in China, in the 14 days prior to symptom onset.
b) Any patient with any acute respiratory illness, whatever the severity, AND with history of at least one of the following exposures in the 14 days prior to illness onset:
   • close contact with a confirmed case of COVID-19, while symptomatic;
   • having shared the same risks of exposure as a confirmed case of COVID-19 (i.e. same history of travel to or residence in China);
   • having worked or attended a health care facility where patients with COVID-19 have been reported;

A **confirmed case** was defined as a possible case with a positive SARS-CoV-2 RT-PCR on respiratory samples, performed by an accredited laboratory.
